# Biochemical Characterization of *Anopheles gambiae* SRPN6, a Malaria Parasite Invasion Marker in Mosquitoes

**DOI:** 10.1371/journal.pone.0048689

**Published:** 2012-11-09

**Authors:** Chunju An, Yasuaki Hiromasa, Xin Zhang, Scott Lovell, Michal Zolkiewski, John M. Tomich, Kristin Michel

**Affiliations:** 1 Division of Biology, Kansas State University, Manhattan, Kansas, United States of America; 2 Department of Entomology, China Agricultural University, Beijing, China; 3 Department of Biochemistry, Kansas State University, Manhattan, Kansas, United States of America; 4 Protein Structure Laboratory, University of Kansas, Lawrence, Kansas, United States of America; Johns Hopkins University, Bloomberg School of Public Health, United States of America

## Abstract

Serine proteinase inhibitors of the serpin family are well known as negative regulators of hemostasis, thrombolysis and innate immune responses. Additionally, non-inhibitory serpins serve functions as chaperones, hormone transporters, or anti-angiogenic factors. In the African malaria mosquito, *Anopheles gambiae s.s*., at least three serpins (SRPNs) are implicated in the innate immune response against malaria parasites. Based on reverse genetic and cell biological analyses, AgSRPN6 limits parasite numbers and transmission and has been postulated to control melanization and complement function in mosquitoes. This study aimed to characterize AgSRPN6 biophysically and determine its biochemical mode of action. The structure model of AgSRPN6, as predicted by I-Tasser showed the protein in the native serpin fold, with three central β-sheets, nine surrounding α-helices, and a protruding reactive center loop. This structure is in agreement with biophysical and functional data obtained from recombinant (r) AgSRPN6, produced in *Escherichia coli*. The physical properties of purified rAgSRPN6 were investigated by means of analytical ultracentrifugation, circular dichroism, and differential scanning calorimetry tools. The recombinant protein exists predominantly as a monomer in solution, is composed of a mixture of α-helices and β-sheets, and has a mid-point unfolding temperature of 56°C. Recombinant AgSRPN6 strongly inhibited porcine pancreatic kallikrein and to a lesser extent bovine pancreatic trypsin in vitro. Furthermore, rAgSRPN6 formed inhibitory, SDS-stable, higher molecular weight complexes with prophenoloxidase-activating proteinase (PAP)1, PAP3, and Hemolymph protein (HP)6, which are required for melanization in the lepidopteran model organism, *Manduca sexta.* Taken together, our results strongly suggest that AgSRPN6 takes on a native serpin fold and is an inhibitor of trypsin-like serine proteinases.

## Introduction

The concept that mosquito immunity could be an important determinant of the infectivity of malaria parasites for different mosquito species was put forward 85 years ago [1]. Even in permissive parasite-vector species combinations, the parasite population undergoes substantial stage-specific losses during its development in the mosquito [2], [3]. Manipulation of the mosquito immune system has been acknowledged as a mechanism to kill malaria parasites within its vector [4]–[9], and the identification of effector mechanisms and molecules that limit parasite development within its mosquito vector remains a research priority.

Proteolytic cascades take a central role in many immune reactions as they amplify the invasion signal and activate various lines of attack against the pathogen. Serine proteinase inhibitors of the serpin family inhibit many of these reactions in arthropods. This includes the hemolymph coagulation cascade in horseshoe crabs [10], proteolytic activation of spätzle and thus the Toll pathway [11]–[15], and proteolytic activation of pro-phenoloxidase (proPO) and as a consequence melanization [16]–[21]. Mosquito serpins (SRPNs) have been shown to control melanization [22]–[25] and host hemostasis during bloodmeal [26], [27]. Additionally, they act as acute response molecules as *An. gambiae* (Ag)SRPN6 and AgSRPN10 are significantly and transiently upregulated during malaria parasite invasion of the midgut [28], [29] and, in case of AgSRPN6, also the salivary glands [30].

Serpins are the largest family of serine proteinase inhibitors and are found in all higher eukaryotes as well as bacteria and viruses (most recently reviewed by [31]). Serpins are metastable proteins that function as structurally conserved suicide substrates [32], [33]. Most serpins inhibit serine proteinases of the chymotrypsin type, but some are cross-class inhibitors that can also target cysteine proteinases [34], [35]. Additionally, some serpins no longer function as proteinase inhibitors but have adopted other roles including hormone transport [36], blood pressure regulation [37], and storage [38]. They can be found intra- as well as extracellularly, and are usually 350–400 amino acid residues long. Although amino acid sequence similarity varies from 17 to 95% across all serpins, key conserved residues facilitate the folding of inhibitory serpins into a metastable conformation typically comprising three β-sheets, eight to nine α-helices, and the solvent-exposed reactive center loop (RCL). Their RCL binds to the active site of the specific target proteinase similar to the binding of a substrate. Upon cleavage of the serpin at its so-called scissile bond (designated P_1_-P_1_′), the serpin undergoes a substantial conformational “stressed-to-relaxed” transition, which covalently traps the target proteinase [33], [39]. Crystal structures of serpins provide additional information into their mechanism of inhibition. So far, more than 80 serpin crystal structures in five distinct conformational states have been solved [39]. To date, protein structures of only a few insect serpins are available. The crystal structure of AgSRPN2 was solved recently in its native conformation [40], and constitutes the first serpin fold described from a dipteran insect. A striking difference between AgSRPN2 and most other native serpins lies in the conformation of the N-terminal hinge region of the RCL, which has partially inserted between two strands of β-sheet A, suggesting an activation mechanism that parallels heparin action on antithrombin III (ATIII) [41]–[43].

A small number of SRPNs have been analyzed for their potential involvement in malaria parasite transmission. AgSRPN6, which is a biomarker for malaria parasite invasion, limits the number of rodent malaria parasites that progress through the midgut and salivary gland epithelium [29], [30]. Depletion of AsSRPN6 by RNAi in susceptible *An. stephensi* leads to a significant increase in the number of developing *P. berghei* oocysts, whereas AgSRPN6 depletion in susceptible *An. gambiae* has no effect on the number of developing parasites but delays the progression of parasite lysis by the complement system and may additionally limit melanization. These observed phenotypic differences are possibly due to changed roles of the respective target serine proteinases in the two mosquito species as both SRPN6 proteins contain identical RCLs. Additionally, knock-down of *AgSRPN6* significantly increases the number of sporozoites reaching the salivary glands. Its endogenous target proteinase(s) await identification, and any western blots performed so far have not revealed any higher molecular bands indicative for serpin-proteinase complexes. It is tempting to speculate that SRPN6 may directly interfere with the function of a parasite proteinase required for epithelial invasion and/or traversal. At least the SRPN6-depletion phenotype would be consistent with such a scenario. However, it remains unclear whether SRPN6 functions as an inhibitory or non-inhibitory serpin.

In the present study, we produced recombinant (r)AgSRPN6 in *Escherichia coli*, characterized its biochemical and physical properties and tested its inhibitory activity using commercial proteinases. We demonstrate that rAgSRPN6 takes on a native conformation and can function as an inhibitor of trypsin-like serine proteinases.

## Materials and Methods

### Protein structure modeling

Structure predictions were performed using the I-Tasser server without additional constraints or templates [44], [45]. The following amino acid sequence (AGAP009212-PA, REF Seq: XP_319990) was submitted online in August 2010: QWNRYYTQAPQARYTPMVRTAQRVALRHSFETDGTPAPSTVRPRPPAPPTNAPSQLPALTPDNDAKISQLVVDFMMRISRTLPQQQSRTELFSPLSIITVANLLFLGSGGSTHEEFGKVLTPSSMNWKRMHQRYGNVLANLMSPEPIDSRRDQWRRQTCPRDDDYEDGEGGPAPKSQVIRVANGIFYQKDLPMRQQYVMLARSLYGALIQPIDPQASAASTALINRWVSDVTAGKIRNMLEGPLSPSSSVVIANALYFKAKWKTQFEPLVTRDAPFFPDGLDGPSYRVKMMSMSGCLPFYRVRDSLDTTIVGLPYRDDTSTMYLIQPANSSRTAIRRLQATLTGKMLDSWISQMKLQSTMVRLPKMHLRNSVDLLQSFQKLGFNSILSPAKSDLSNMIDSSSSAGPKPYVNQILHKLDLTIDEEGTEGAAATSALVDRIGSQRQFNGNAPFLIYLRHDATGLPLFYGPIFDPR

### Expression and purification of recombinant proteins

To express full-length rAgSRPN6, the entire *AgSRPN6* coding region, excluding the signal peptide, was amplified by PCR using SRPN6.3/pGEMT-easy plasmid [29] as template with the following primer pair: S6F: 5′-TACC*ATG*GGCCATCATCATCATCATCACGGCCAGTGGAATCGGTACTACA-3′ (*Nco*I restriction site underlined, start codon italicized, and codons for six histidine residues double-underlined); S6R: 5′-CGAAGCTTTTAGCGCGGATCGAATATCGGCCCG-3′ (*Hind*III restriction site underlined). The PCR product was digested with *Nco*I and *Hind*III, and inserted into the same restriction sites in the expression vector pET-28a (Novagen EMD, Rockland, MS, USA). N-terminal His-tagged rSRPN6 protein was expressed and purified as reported previously [24].

For the generation of antibodies against AgSRPN6 a cDNA fragment encoding the C-terminal 201 amino acid residues of the protein was amplified by PCR using SRPN6.3/pGEMT-easy plasmid [29] as template with the following primers: S6AgF: 5′-TACC*ATG*GGCCATCATCATCATCATCACGGCGATGCACCCTTCTTCCCGGA-3′ (*Nco*I restriction site underlined, start codon italicized, codons for six histidine residues double-underlined); and primer S6R. The PCR product was digested and subcloned into vector pET28a (Novagen) as described above. Recombinant N-terminally His-tagged SRPN6Ag was expressed in a one liter culture of *E. coli* strain BL21 with 0.1 mM of isopropyl β-D-thiogalactoside for 5 h at 20°C, 250 rpm. SRPN6Ag was expressed in an insoluble form and therefore was purified under denaturing conditions by nickel-nitrilotriacetic acid agarose affinity chromatography (Qiagen, Valencia, CA, USA) [46]. An aliquot of the purified recombinant SRPN6Ag (1.5 mg) was further resolved by preparative SDS-PAGE and used for polyclonal rabbit antiserum production (Cocalico Biologicals, Reamstown, PA, USA).

Recombinant *M. sexta* proHP6 (proHP6Xa), in which the cleavage activation site LDLH92 was replaced with IEGR92 to permit its activation by bovine Factor Xa, was prepared and activated by Factor Xa as described previously [47]. Recombinant active *M. sexta* PAP1 and PAP3 were kindly provided by Dr. Haobo Jiang (Oklahoma State University) and Dr. Maureen Gorman (Kansas State University), respectively.

### Proteinase inhibition assays

To measure the inhibitory activities of AgSRPN6, the recombinant protein was incubated with various commercial proteinases at different molar ratios in 0.1 M Tris, pH 8.0. 3 µg of BSA was included in each reaction. After incubation at room temperature for 10 min, 200 µl of appropriate chromogenic substrate solution (100 µM in 0.1 M Tris-HCl, 0.1 M NaCl, 5 mM CaCl_2_, pH 8.0) was added, and residual enzyme activity was immediately measured at A405 in a PowerWave XS microplate reader (Bio-Tek, Winooski, VT, USA). One unit of amidase activity was defined as ΔA405 = 0.001/min. The following proteinases and their artificial substrate were used: human leukocyticcathepsin G (200 ng) and N-succinyl-Ala-Ala-Pro-Phe-p-nitroanilide; bovine pancreatic α-chymotrypsin (120 ng) andN-succinyl-Ala-Ala-Pro-Phe-p-nitroanilide; porcine pancreatic elastase (2 µg) and N-succinyl-Ala-Ala-Pro-Leu-p-nitroanilide; porcine pancreatic kallikrein (700 ng) and D-Phe-L-Pro-L-Arg-p-nitroanilide; human serum plasmin (200 ng) and D-Phe-L-Pro-L-Arg-p-nitroanilide; proteinase K from the fungus *Tritirchium album* (40 ng) and N-succinyl-Ala-Ala-Pro-Phe-p-nitroanilide; subtilisin Carlsberg from *Bacillus licheniformis* (50 ng) and N-benzoyl-Phe-Val-Arg-p-nitroanilide; human serum thrombin (30 ng) andN-benzoyl-Phe-Val-Arg-p-nitroanilide; bovine pancreatic trypsin (5 ng) and N-acetyl-Ile-Glu-Ala-Arg-p-nitroanilide (Biochemistry Core Facility, Kansas State University). All proteinases and substrates were purchased from Sigma-Aldrich (St. Louis, MO, USA), with the exception of proteinase K (Promega, Madison, WI, USA) and N-acetyl-Ile-Glu-Ala-Arg-p-nitroanilide, which was synthesized in-house.

### Sedimentation velocity

Purified intact rAgSRPN6 was dialyzed against 20 mM Tris and 250 mM NaCl, pH 8.0 at 4°C for 24 h with one buffer exchange to reach a concentration of 1.0 mg/ml. Same dialysis buffer was used as a blank. Sedimentation velocity runs were performed in an Optima XL-I analytical ultracentrifuge (Beckman Coulter, Brea, CA, USA) at 49,000 rpm, 25°C in an An-60 Ti rotor. Sedimentation was monitored at 280 nm using double sector cells with a final loading of 400 µl per well. Acquired data were analyzed using DCDT+ software version 1.16, and sedimentation coefficients were calculated using g(s*) fitting function in the DCDT+ software [48].

### Circular dichroism

To investigate the effect of pH on the CD spectrum of rAgSRPN6, purified protein was dialyzed as above and the pH was adjusted to 5.0 or 11.0 by stepwise addition of either 0.1 M HCl or 1.0 M NaOH. Samples were centrifuged at 13,000 rpm for 5 min to remove any pellet resulting from pH change. Far-UV CD spectra (190–260 nm) were recorded at 25°C with a Jasco J-815 CD Spectrometer (Jasco Corporation, Tokyo, Japan). A cell of 0.2 mm path length was used in the scanning from 260 nm to 190 nm with a scan rate of 50 nm/min. Spectra were the averaged over ten scans. All CD spectra were smoothed and corrected by background subtraction for the spectrum obtained with buffer alone. Data were expressed as molar ellipticity per mean residue ([*θ*] = degree • cm^2^• dmol^−1^) with a calculated mean residue molecular weight of 111.78, based on a molecular weight of 53,877 Da and 482 amino acid residues.

### Differential scanning calorimetry

To check the thermal stability of rAgSRPN6, purified protein (1.0 mg/ml) was dialyzed as above, degassed, and loaded into VP-DSC MicroCalorimeter (MicroCalTM Inc.). Temperature was scanned at 1°C/min from 20°C to 85°C. Heat capacity, Cp (kcal/mol/°C), was plotted after background subtraction of a blank experiment without protein.

### Formation of SDS-stable complexes between rSRPN6 and proteinase

Active *M. sexta* PAP1 (15 ng) or PAP3 (20 ng) was incubated with 200 ng of purified rAgSRPN6 (molar ratio of 1∶10 for proteinase/serpin) at room temperature for 10 min and then treated with SDS reducing sample buffer at 95°C for 5 min. The reaction mixtures were resolved by 10% SDS -polyacrylamide gel electrophoresis and subjected to Western blot analysis using 1∶2,000 diluted antiserum against *M. sexta* PAP-1 [49], PAP-3 [18] or AgSRPN6 as primary antibodies. 15 ng of recombinant proHP6Xa was produced and activated as described previously [47], and mixed with purified rAgSRPN6 at a molar ratio of 1∶10 (HP6/SRPN6). In control samples, proHP6Xa or factor Xa were omitted from the mixture. After incubation at room temperature for 10 min, the reaction mixtures were analyzed by immunoblotting using antiserum against *M. sexta* HP6 [50] and AgSRPN6.

### ProPO activation assays

To investigate the effect of rAgSRPN6 on proPO activation, cell-free hemolymph was collected from day-2 fifth instar *M. sexta* larvae as described previously [19]. 200 ng of recombinant rAgSRPN6 (or rAgSRPN7 as negative control) was mixed with 1 µl of hemolymph for 10 min at room temperature. Subsequently, 1 µl of *Micrococcus luteus* (10 µg/µl in sterile water, Sigma) was added to trigger proPO activation in the hemolymph. After incubation for 10 min at room temperature, PO activity was measured using dopamine as a substrate. One unit of PO activity was defined as the amount of enzyme producing an increase in absorbance (ΔA470) of 0.001 per min.

## Results and Discussion

*AgSRPN6* mediates mosquito innate immune response against malaria parasites with unknown mechanisms [29], [30]. To gain further insight into AgSRPN6's mode of action, we investigated its structural, biophysical, and biochemical properties using modeling, circular dichroism, differential scanning calorimetry, and amidase activity assays.

### Protein Structure Prediction of SRPN6

**Figure 1:**
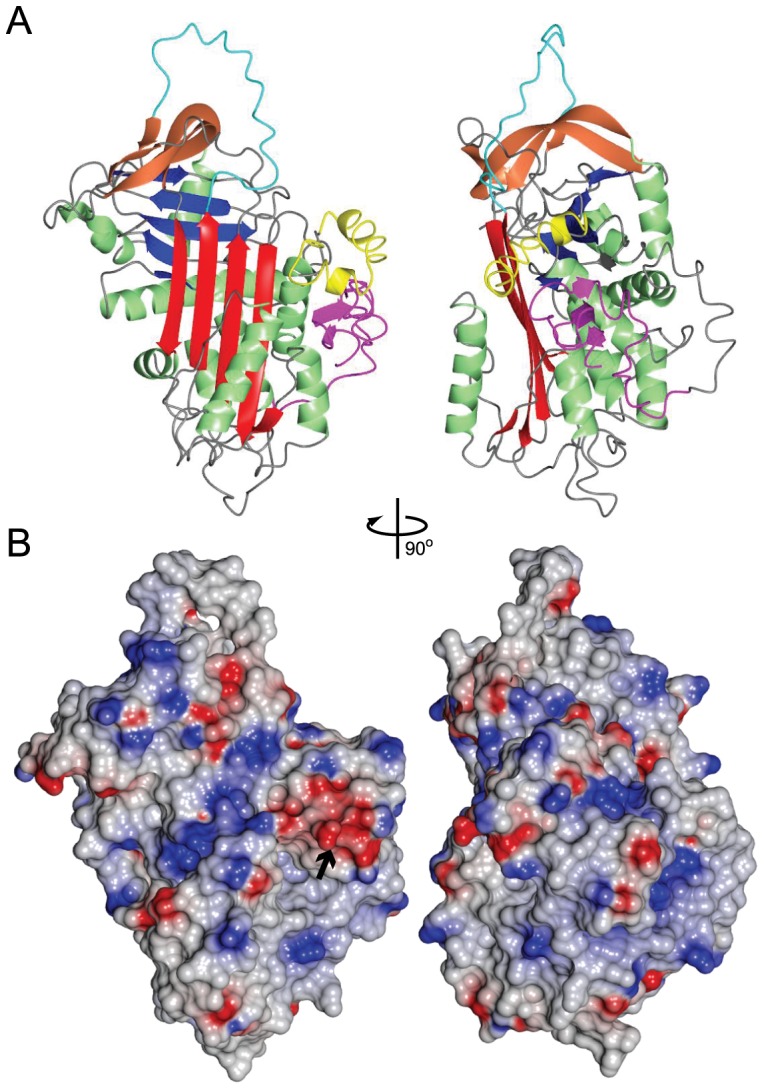
Structure model of AgSRPN6. Ribbon structure (**A**) and electrostatic surface representation (**B**) of the I-Tasser model with the highest C-score (−0.77). Highlighted in pink are the first 41 amino acids of SRPN6, which constitute an N-terminal extension unique to AgSRPN6. This extension is in close proximity to a 27 amino acid long insertion located between helix D and strand 2A (yellow). This insertion forms two short alpha helices and contains a stretch of acidic residues, which form a negatively charged surface area, highlighted by a black arrow. Bright red, sheet A; blue, sheet B; tan, sheet C; green, α-helices; cyan, RCL. Blue surface, negative charges; red surface, positive charges.

Since no structure is currently available for AgSRPN6, a predicted 3-dimensional model was computed using the I-TASSER server [44], [45], which yielded a C-score of −0.77 and TM score of 0.62 (0.14) for the top model. The predicted structure is similar to the native fold of other serpins consisting of three β-sheet domains, 8 α-helices and a RCL (Figure 1). A comparison with other structures suggested that SRPN6 overall shares highest similarity with human antithrombin III (PDB: 1E03, chain I) with a calculated RMSD of 1.47 Å. Similarity to AgSRPN2, the only mosquito serpin for which the protein structure was solved by X-ray crystallography, is lower, with a calculated RMSD of 1.69 Å (PDB: 3PZF, [40]). Similarity to *M. sexta* Serpin 1K (PDB: 1SEK, [51]) is comparable with an RMSD of 1.67 Å, and likewise to *T. molitor* Serpin48 (PDB: 3OZQ, [52]) with an RMSD of 1.63 Å.

Sequence comparison between AgSRPN6 and other arthropod serpins had previously revealed an insertion of 27 amino acids (aa 146–174) between helix D and strand 2A. This insertion is restricted to the members of a mosquito-specific expansion cluster including SRPN4, 5, 6, 16 and 19 [22], [53]. The structure of this insertion is predicted to form two one turn α-helices above and perpendicular to helices D and E (Fig. 1A, maroon). An additional feature of SRPN6 is a 41 amino acid long C-terminal extension. This extension is predicted to form a short β-sheet, which runs parallel to helix D (Figure 1A, pink). Several hydrogen bonds are predicted between the insertion and N-terminal extension, specifically between amino acid residues 19/162, 20/164, 20/158, 21/151, and 23/148, suggesting that these two structural features interact and stabilize each other. Additionally, the insertion contains several aspartic acid residues that form a negatively charged surface area which could be an important binding site. Relatively few serpins with either insertions within the core serpin fold, or N-terminal extensions, can be found in the literature. An interesting example is the *D. melanogaster* Necrotic serpin, which has an N-terminal extension that is cleaved on immune-challenge. This cleavage modifies the affinity of the Nec serpin for Elastase, and presumably it's in vivo target proteinase [54]. If the N-terminal extension in AgSRPN6 is cleaved *in vivo* is currently unknown.

### Production of recombinant *An. gambiae* SRPN6

**Figure 2:**
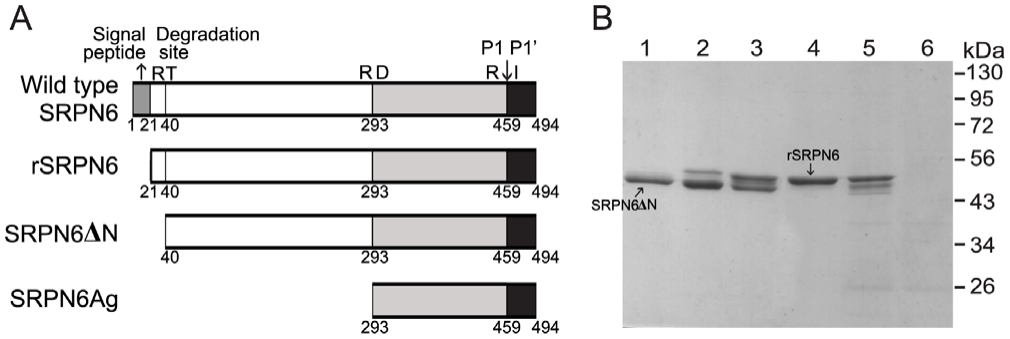
Production of rAgSRPN6. (A) Schematic representation of rAgSRPN6. Letters above the boxes represent the amino acid residues and the numbers underneath indicate their positions in wild type AgSRPN6. Predicted P1-P1′ cleavage sites are indicated by arrows. (B) SDS-PAGE analysis of purified rAgSRPN6. Fraction #16, #18, #20, and #22 from SP-sepharose cation exchange chromatography (1 µg/sample, *Lane* 1–4 respectively) were separated by 10% SDS-PAGE followed by commassie blue staining. *Lane* 5: 1 µg of rAgSRPN6 (faction #22) was incubated with 3×10^9^ *E. coli* XL1-blue cells at room temperature for 15 min and subjected to SDS-PAGE. *Lane* 6: 3×10^9^ *E. coli* XL1-blue cells.

To further characterize AgSRPN6, we expressed recombinant protein, excluding signal peptide, in *E. coli* (Fig. 2A). Primary trials indicated almost all rAgSRPN6 was insoluble after expression at 37°C. Therefore, large scale production of soluble, rAgSRPN6 was expressed at a lower temperature of 20°C with low speed shaking at150 rpm. The N-terminally His-tagged recombinant SRPN6 was purified by Ni-NTA affinity chromatography, followed by SP-sepharose cation exchange chromatography. Approximately 20 mg of rAgSRPN6 could be obtained from 1.5 L of bacterial culture. SDS-PAGE analysis of recombinant SRPN6 indicated purified SRPN6 was of high purity (Fig. 2B) with the apparent molecular weight of 54 kDa. In addition to this major band, another band at 53 kDa position appeared in some fractions. N-terminal sequencing results confirmed that this band was an N-terminally truncated form of rAgSRPN6 (SRPN6ΔN) degraded after Arg^40^, different from predicted P1 cleavage site Arg^459^ [29]. Incubation of full-length rSRPN6 with *E. coli* cell lysates resulted in the appearance of this truncated band (Fig. 2B). Therefore, appearance of truncated forms is likely due to the cleavage by unknown *E. coli* proteinases and therefore an artifact of the isolation procedure. Full-length rAgSRPN6 form was separated from the truncated form by SP-sepharose cation exchange chromatography, and used for all subsequent analyses.

Additionally, a C-terminal rAgSRPN6 form, SRPN6Ag, covering the last 201 amino acid residues of full-length SRPN6 was produced. This polypeptide was insoluble, and purified under denaturing conditions by Ni-NTA and preparative SDS-PAGE. SRPN6Ag was used successfully to produce polyclonal antibodies against AgSRPN6 (Fig. S1).

### Biophysical and structural parameters of recombinant SRPN6

**Figure 3:**
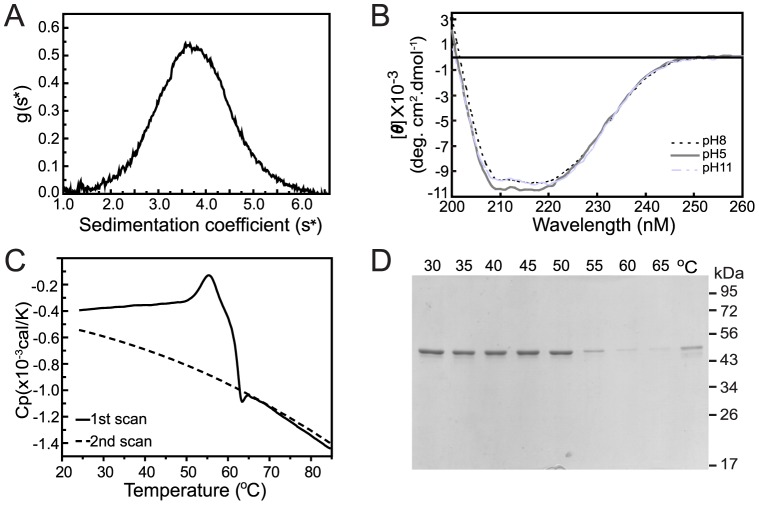
Biophysical and structural parameters of rAgSRPN6. (**A**) Sedimentation velocity analysis of rAgSRPN6. Dialyzed rAgSRPN6 (1 mg/ml) was evaluated by analytical ultracentrifugation. Data were analyzed using DCDT+ software version 1.16 [48]. (**B**) Far-UV CD spectra of rAgSRPN6. The protein was dissolved in 20 mM Tris-HCl buffer containing 250 mM NaCl with pH of 8.0 (—) or 5.0 (—) or 11.0 (– -). [*θ*] represents mean residue elliplicity defined in “Material and methods”. (**C**) Calorimetric scans of rAgSRPN6. Samples were prepared by dialysis against 20 mM Tris and 250 mM NaCl, pH 8.0 and analyzed by DSC. Traces were baseline-corrected by subtraction of a scan of buffer. (**D**) SDS-PAGE analysis of purified rAgSRPN6 heated at different temperatures. One µg of purified rAgSRPN6 was heated at 30, 35, 40, 45, 50, 55, 60, and 65°C for 5 min, respectively, and centrifuged at 13,000 rpm for 5 min. The supernatants were separated by 10% SDS-PAGE followed by commassie blue staining (*Lane* 1–8 respectively). *Lane* 9: Supernatant from 1 µg of rAgSRPN6 analyzed after DSC scan.

To gain information on the structural parameters of rAgSRPN6 in solution, we performed sedimentation velocity experiments by analytical ultracentrifugation (Fig. 3A). The sample contained a single molecule species with the sedimentation coefficient of 3.74 Svedberg units and the molecular mass was calculated as 53.6 kDa. This is approximately equal to the molecular weight (53.9 kDa) calculated from amino acid sequences of rAgSRPN6. This result indicates that rAgSRPN6 exists predominantly as a monomer in solution and is not prone to form dimers or polymers as previously reported for mutant forms of α1 antitrypsin [55] or neuroserpin [56].

Native and rAgSRPN6 have a calculated pI of 9.6, which is substantially more basic compared to most *Anopheles* serpins with calculated pIs between 5 and 6. To test whether pH influences the conformation of the protein we determined the far ultraviolet (UV) circular dichroism spectra of recombinant SRPN6 at different pH. The canonical native serpin fold is composed of a mixture of α-helices, β-sheets, β-turns, and loops, presented as a minimum peak at 222 nm preceded by a shoulder at 208 nm in the far-UV CD spectra [57]–[59]. The far UV CD spectrum of SRPN6 is highly similar to that of reported serpins (Fig. 3B). All spectra demonstrated broad negative bands in the wavelength range of 208–222 nm, with [θ]≈−9900 deg. cm^2^. dmol^−1^ for SRPN6 at pH 8.0 and 11.0, and [θ]≈−10500 deg. cm^2^. dmol^−1^ for SRPN6 at pH 5.0. The general shape of the CD spectra at pH 5.0, 8.0 and 11.0 was approximately indistinguishable, suggesting that recombinant SRPN6 kept the same high degree of secondary structure under either acid or basic conditions.

The native conformation of serpins, which generally represents the active conformation of the protein, is the least stable conformation. This is reflected in an average midpoint unfolding temperature (T_m_) of the native form of about 58°C, while serpin molecules in the latent conformation usually display melting temperatures of 85–90°C ([60], and references within). The thermal stability of recombinant SRPN6 was analyzed by Differential scanning calorimetry (DSC). As shown in Fig. 3C, rAgSRPN6 displayed a T_m_ of 56°C. The DSC thermogram was distorted at temperatures higher than the T_m_, most likely due to aggregation of rAgSRPN6 at higher temperatures. No heat capacity peak was observed during the second DSC scan, suggesting that the SRPN6 structure was destroyed completely when the temperature increased above 56–58°C and denaturation was irreversible even when the temperature decreased below T_m_. Accordingly, rAgSRPN6 protein concentration in solution decreased around 55°C (Fig. 3D), due to protein aggregation and precipitation. Taken together, the DSC analysis results are consistent with rAgSRPN6 protein taking on a native conformation in solution, with little or no propensity for a latent conformation.

### Inhibitory activity of recombinant rSRPN6

**Figure 4:**
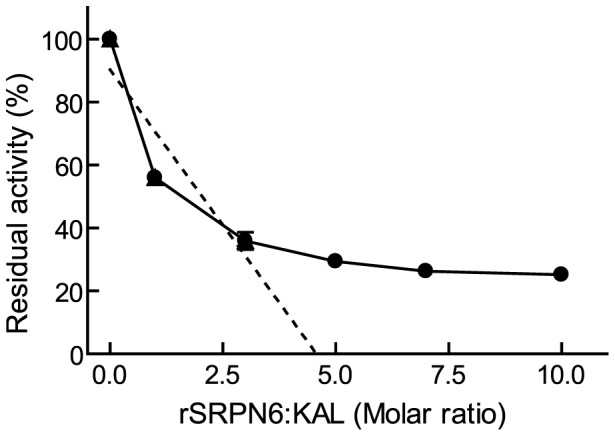
Stoichiometry of inhibition of kallikrein by rAgSRPN6. Recombinant AgSRPN6 was incubated with porcine kallikrein at different molar ratios for 10 min at room temperature. The residual amidase activity was measured using FPR*p*Na as substrate.

The functionality of purified rAgSRPN6 was assayed in commercial proteinase inhibition tests, in which the enzymatic activity of proteinases was monitored using chromogenic substrates. The tested proteinases included mammalian digestive enzymes (trypsin, chymotrypsin), mammalian tissue proteinases with distinct functions (elastase, kallikrein), several serine proteinases from human blood (cathepsin G, plasmin, thrombin) and subtilisin-like proteinases (proteinase K, subtilisin *Carlsberg*). SRPN6 has the basic residue Arginine at the predicted P1 site, suggesting it as a potential inhibitor of trypsin-like serine proteinases, which prefer Arginine or Lysine at the N-terminal residues of the target peptide bond. The results are summarized in Table 1. As expected based on the substrate specificity of the proteinase, rAgSRPN6 had no inhibitory activity against cathepsin G, chymotrypsin, proteinase K, or elastase. Plasmin, subtilisin *Carlsberg*, thrombin, trypsin, and kallikrein prefer an Arg, or Lys at the P1 site, and therefore are potential inhibitory targets of SRPN6. However, rAgSRPN6 did not inhibit Plasmin, subtilisin *Carlsberg*, thrombin, and only weakly inhibited trypsin by ∼13% at a serpin/trypsin molar ratio of 10∶1. rSRPN6 inhibited kallikrein most efficiently among all tested commercial proteinases, with 44% and 75% inhibition at rAgSRPN6/kallikrein ratio of 1∶1 and 10∶1, respectively. Kallikrein activity decreased linearly as rAgSRPN6 concentration increased (Fig. 4). Therefore, rAgSRPN6 (and most likely the native protein) is a functional serine proteinase inhibitor. Its inhibitory specificity is distinct from that of aFXa [61] and SRPN2 [24], the only other mosquito serpins that have been characterized biochemically.

**Table 1:** Proteinase inhibition assays with rAgSRPN6.

Predicted cleaved site	rSRPN6∶ Proteinase	Cath G	CHY	ELA	KAL	PLAS	Pro K	Sub C	THRMB	TRYP
LVDR∥ IGSQ	1∶1	–	–	–	44.0±1.7	–	–	–	–	–
	10∶1	–	–	–	74.9±1.0	–	–	–	–	13.0±5.1

Human cathepsin G (Cath G), bovine chymotrypsin (CHY), porcine elastase (ELA), porcine kallikrein (KAL), Human plasmin (PLAS), Fungus protease K (ProK), *Bacilus licheniformis* Subtilisin Carlsberg (SubC), human thrombin (THRMB), and bovine trypsin (TRYP) were incubated with purified rAgSRPN6 and assayed for residual enzyme activity. “–”, inhibition <10%. Values represent mean percent inhibition ± S.D. (n = 3).

### Recombinant SRPN6 forms SDS-stable complexes with insect serine proteinases

**Figure 5:**
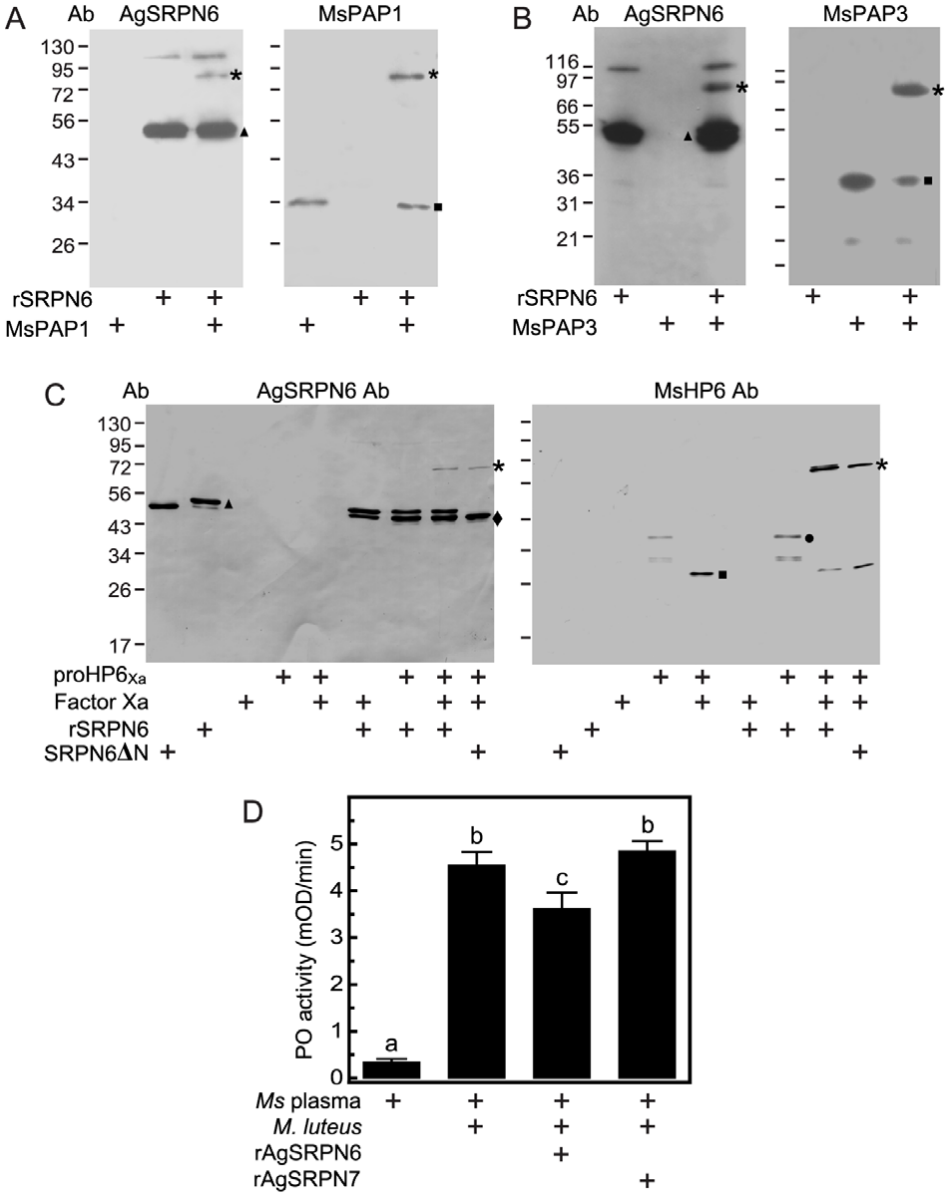
SDS-stable complex formation between rAgSRPN6 and *M. sexta* PAP1, PAP3, and HP6. Purified rAgSRPN6 was incubated with *M. sexta* PAP1 (**A**) or PAP3 (**B**) for 10 min at room temperature. The mixtures were subjected to SDS-PAGE under reducing conditions, and the complex was detected by immunoblotting using antisera against either AgSRPN6 (*left* panel) or MsPAP (*right* panel). (C) ProHP6_Xa_ was activated by factor Xa and then incubated for 10 min at room temperature with rAgSRPN6 at a molar ratio of 10∶1 (rAgSRPN6∶proHP6_Xa_). In control reactions, proHP6_Xa_ or factor Xa were omitted. The samples were subjected to SDS-PAGE and immunoblot analysis using antiserum against AgSRPN6 (*left* panel) or MsHP6 (*right* panel). Size and positions of molecular mass standards are indicated to the *left* of the blots. Asterisks, rAgSRPN6-proteinase complex; triangles, non-complexed rAgSRPN6; diamond; SRPN6ΔN; circles, proHP6_Xa_ zymogen; squares, catalytic domain of serine proteinase. (D) Inhibition of ProPO activation in *M. sexta* hemolymph by rAgSRPN6. 1 µl of cell-free hemolymph was incubated with 200 ng of rAgSRPN6 or rAgSRPN7. 10 min later, ProPO activation was triggered by the addition of *M. luteus* to a final concentration of 3 µg/µl. PO activity (mean±S.D., n = 3) was measured after 10 min in a spectrophotometer as described in “Material and Methods”. Letters above columns indicate significance levels of *P*<0.0001 (One way ANOVA, Newman-Keuls multiple comparison test).

Knockdown of *AgSRPN6* increases the number of melanized ookinetes in the refractory L3–5 strain as well as in CTL4-depleted mosquitoes [29]. One potential explanation for these observations is that AgSRPN6 under normal physiological conditions suppresses melanization of parasites, by inhibiting one or more PAPs and thus preventing activation of proPO and melanization. To test this hypothesis, we made use of the *M. sexta* melanization cascade, which is established *in vitro* and *ex vivo*. A characteristic feature of a serpin-proteinase reaction is the formation of a high molecular weight, SDS-stable complex [62]. We made use of *M. sexta* serine proteinase PAP1, PAP3, and HP6 to investigate whether rAgSRPN6 can form such complex *in vitro*. *M. sexta* PAP1 antibodies recognized a 34 kDa band corresponding to the catalytic domain of PAP1 but not rAgSRPN6 (Fig. 5A, right panel). After the two proteins were incubated together, the 34 kDa catalytic domain became fainter and a new immunoreactive band migrated to the ∼80 kDa position anticipated for a complex of rAgSRPN6 and PAP1 catalytic domain. This ∼80 kDa band was also detected by AgSRPN6 antibodies produced against the last 201 amino acid residues of AgSRPN6 (Fig. 5A, left panel), indicating that it was composed of these two proteins. Mass-spec analysis confirmed the presence of rAgSRPN6 and PAP1 in the ∼80 kDa band (data not shown). Surprisingly, the previously published antiserum raised against soluble full-length rAgSRPN6 [29] did not recognize this complex (Fig. S2).

Similar results were obtained for the interaction between rAgSRPN6 and PAP3 (Fig. 5B). The interaction of rAgSRPN6 and PAP3 resulted in the formation of an ∼80 kDa complex under reducing conditions, which was absent from the control lanes with only PAP3 or SRPN6 alone. This complex was recognized by both rAgSRPN6 antibodies and *M. sexta* PAP3 antibodies (Fig. 3B) indicating that SRPN6 can also form a covalent complex with PAP3.

Consistent with previous report [47], anti-HP6 antiserum recognized the 39 kDa proHP6 zymogen and a 33 kDa truncated HP6 in the absence of factor Xa, and after activation with factor Xa, a 29 kDa band corresponding to the catalytic domain of HP6_Xa_was detected by the HP6 antiserum (Fig. 5C, right panel).When rAgSRPN6 was mixed with active HP6_Xa_, a new immunoreactive band at ∼72 kDa position (the expected size for a SRPN6:HP6_Xa_ complex) was observed. This band was also recognized by AgSRPN6 antiserum (Fig. 5C, left panel). The incubation of truncated AgSRPN6 (SRPN6ΔN) and active HP6_Xa_ also resulted in the appearance of 72 kDa band recognized by both antibodies (Fig. 3C), indicating truncated SRPN6 retains the ability to form SDS-stable inhibitory complexes with proteinases.

The high *M*_r_, covalent complex formation between rAgSRPN6 and PAP1, PAP3, and HP6 supports the hypothesis that AgSRPN6 can regulate melanization as these three proteinases are critical components of the proPO activation pathway in *M. sexta* [18], [47], [49]. To further test this hypothesis, we investigated AgSRPN6's effect on proPO activation *ex vivo*. Naïve hemolymph from day 2 fifth instar *M. sexta* larvae has low basal PO activity, and exposure of *M. luteus* significantly increased the PO activity of hemolymph (Fig. 5D and [63]). Pre-incubation of hemolymph with rAgSRPN6 significantly decreased PO activity (Fig. 3D). Therefore, rAgSRPN6 had inhibitory effect on the proPO activation in *M. sexta* hemolymph, most likely by inhibiting PAP1, PAP3 and HP6 in the hemolymph. These findings are consistent with our working hypothesis that one function of AgSRPN6 is regulating melanization *in vivo*.

Recently, the first PAP from *An. gambiae* was identified and characterized biochemically. AgCLIPB9 is part of a regulatory unit that controls melanization in adult females, by functioning as a PAP. However, active recombinant CLIPB9_Xa_ did not form inhibitory complexes with rAgSRPN6 (Fig. S3), and its amidase activity was unaltered in the presence of rSRPN6. However, additional PAPs are likely to be present in the hemolymph of mosquitoes, which may be inhibited by SRPN6. Antibodies against the last 201 amino acid residues of AgSRPN6 that were created for this project do recognize the native state of the serpin and also bind to serpin-proteinase complexes formed with heterologous proteinases *in vitro*. However, no SRPN6-proteinase complexes were identified in hemolymph, supernatants of the hemocyte-like 4a3B *An. gambiae* cell line [64], and *P. berghei*-infected epithelia (data not shown), suggesting that the endogenous target(s) of AgSRPN6 are either absent or present in undetectable concentrations under physiological conditions.

## Conclusions

Structural modeling of AgSRPN6 suggested that this serpin can take on the metastable classical serpin fold, which is supported by the biophysical data presented in this study. The biochemical characterization confirmed that rAgSRPN6 is indeed an inhibitor of trypsin-like serine proteinases. Additionally, this study provides the first biochemical evidence that AgSRPN6 controls the melanization response by inhibiting proteinases of the pro-phenoloxidase activation pathway. It is noteworthy that we found differences between the abilities of different antisera to recognize higher molecular weight complexes between rAgSRPN6 and target proteinases. The new antiserum raised against a C-terminal polypeptide of AgSRPN6 is a key step towards future identification of the cognate proteinase targets and will allow the identification of novel components of the proPO and or complement pathway in mosquitoes.

## Supporting Information

Figure S1 **SDS-PAGE of recombinant SRPN6Ag.** The last 201 amino acid residues of SRPN6 (SRPN6Ag) were expressed in *E. coli* and purified under denaturing conditions. 10 µl of elution fraction #1-#5 (*Lane* 1–5, respectively) were separated by 10% SDS-PAGE followed by Coomassie Blue staining.
(TIF)

Figure S2 **Detection of SDS-stable complex formation between** ***An. gambiae*** **SRPN6 and** ***M. sexta*** **PAP1.** Purified recombinant SRPN6 (200 ng) was incubated with *M. sexta* PAP1 (15 ng) at room temperature for 10 min. The mixtures were subjected to SDS-PAGE and immunoblot analysis using previously published antisera against either full-length rSRPN6 (*left* panel) or MsPAP1 (*right* panel). The band with apparent molecular weight of 80 kDa (the expected size for a SRPN6:PAP1 complex) was recognized by MsPAP1 antibodies (*right* panel), but not by antibodies against recombinant full length SRPN6 (*left* panel). However, this band was detected by the antibodies against SRPN6Ag (Fig. 5A–5C). Asterisks, SRPN6-PAP1 complex; triangles, non-complexed rSRPN6; squares, active PAP1.
(TIF)

Figure S3 **No covalent complex formed between rSRPN6 and serine proteinase CLIPB8 and CLIPB9.** Recombinant SRPN2 was incubated with Factor Xa-activated CLIPB9Xa. Western blot analysis was performed using rabbit anti-SRPN6 antibodies against recombinant SRPN6Ag. No complex band except recombinant rSRPN6 was detected. A smaller band was detected after incubation with FactorXa, suggesting that rSRPN6 constitutes a substrate for this proteinase. Triangles, non-complexed full-length rSRPN6; diamond, cleaved rSRPN6.
(TIF)
