# Supplementary figures and images for: Biochemical Characterization of Anopheles gambiae SRPN6, a Malaria Parasite Invasion Marker in Mosquitoes
Source: PLoS One. 2012 Nov 9;7(11):e48689. doi: 10.1371/journal.pone.0048689 (PMC3494705; doi:10.1371/journal.pone.0048689)

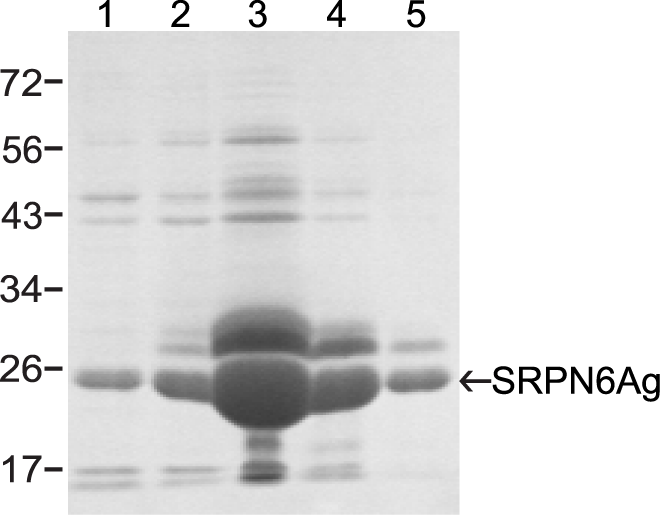

Supplement: Figure S1 — SDS-PAGE of recombinant SRPN6Ag. The last 201 amino acid residues of SRPN6 (SRPN6Ag) were expressed in E. coli and purified under denaturing conditions. 10 µl of elution fraction #1-#5 (Lane 1–5, respectively) were separated by 10% SDS-PAGE followed by Coomassie Blue staining. (TIF) [file pone.0048689.s001.tif]

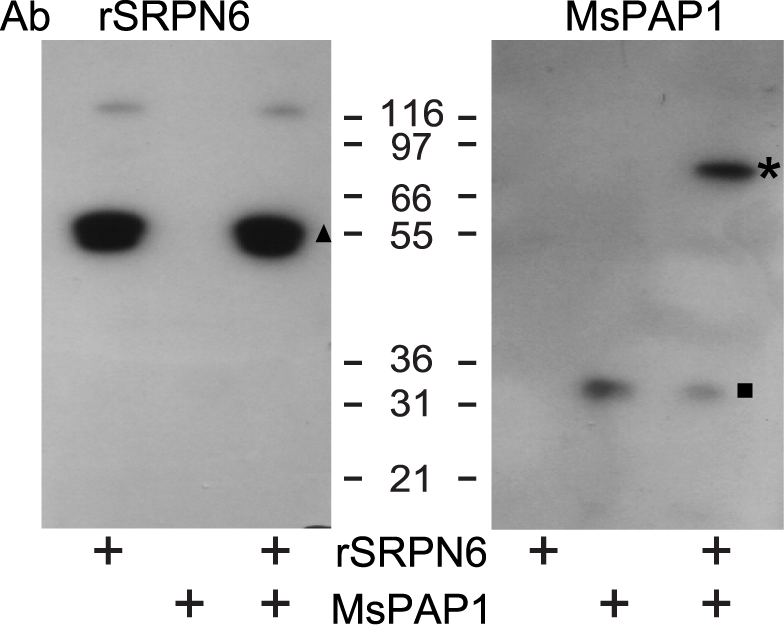

Supplement: Figure S2 — Detection of SDS-stable complex formation between An. gambiae SRPN6 and M. sexta PAP1. Purified recombinant SRPN6 (200 ng) was incubated with M. sexta PAP1 (15 ng) at room temperature for 10 min. The mixtures were subjected to SDS-PAGE and immunoblot analysis using previously published antisera against either full-length rSRPN6 (left panel) or MsPAP1 (right panel). The band with apparent molecular weight of 80 kDa (the expected size for a SRPN6:PAP1 complex) was recognized by MsPAP1 antibodies (right panel), but not by antibodies against recombinant full length SRPN6 (left panel). However, this band was detected by the antibodies against SRPN6Ag (Fig. 5A–5C). Asterisks, SRPN6-PAP1 complex; triangles, non-complexed rSRPN6; squares, active PAP1. (TIF) [file pone.0048689.s002.tif]

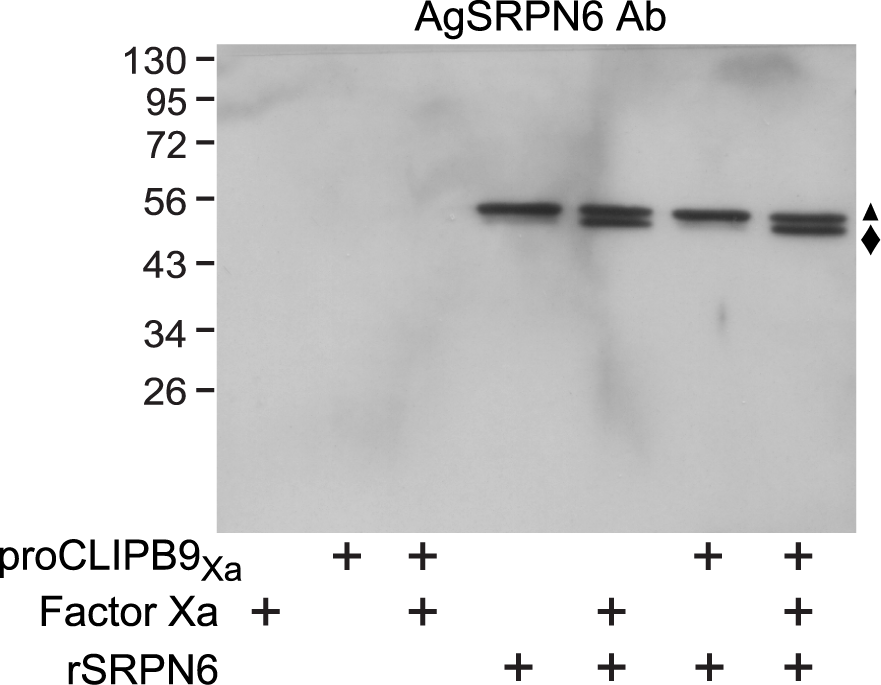

Supplement: Figure S3 — No covalent complex formed between rSRPN6 and serine proteinase CLIPB8 and CLIPB9. Recombinant SRPN2 was incubated with Factor Xa-activated CLIPB9Xa. Western blot analysis was performed using rabbit anti-SRPN6 antibodies against recombinant SRPN6Ag. No complex band except recombinant rSRPN6 was detected. A smaller band was detected after incubation with FactorXa, suggesting that rSRPN6 constitutes a substrate for this proteinase. Triangles, non-complexed full-length rSRPN6; diamond, cleaved rSRPN6. (TIF) [file pone.0048689.s003.tif]
